# Predicting Visual Acuity in Patients Treated for AMD

**DOI:** 10.3390/diagnostics12061504

**Published:** 2022-06-20

**Authors:** Beatrice-Andreea Marginean, Adrian Groza, George Muntean, Simona Delia Nicoara

**Affiliations:** 1 Department of Computer Science, Technical University of Cluj-Napoca, 400114 Cluj-Napoca, Romania; andreea.beatrice2@yahoo.com; 2 Department of Ophthalmology, “Iuliu Hatieganu” University of Medicine and Pharmacy, 400012 Cluj-Napoca, Romania; georgemuntean99@gmail.com (G.M.); simonanicoara1@gmail.com (S.D.N.); 3 Emergency County Hospital, 400347 Cluj-Napoca, Romania

**Keywords:** diagnosis of retinal conditions, OCT, predicting visual acuity, machine learning

## Abstract

The leading diagnostic tool in modern ophthalmology, Optical Coherence Tomography (OCT), is not yet able to establish the evolution of retinal diseases. Our task is to forecast the progression of retinal diseases by means of machine learning technologies. The aim is to help the ophthalmologist to determine when early treatment is needed in order to prevent severe vision impairment or even blindness. The acquired data are made up of sequences of visits from multiple patients with age-related macular degeneration (AMD), which, if not treated at the appropriate time, may result in irreversible blindness. The dataset contains 94 patients with AMD and there are 161 eyes included with more than one medical examination. We used various techniques from machine learning (linear regression, gradient boosting, random forest and extremely randomised trees, bidirectional recurrent neural network, LSTM network, GRU network) to handle technical challenges such as how to learn from small-sized time series, how to handle different time intervals between visits, and how to learn from different numbers of visits for each patient (1–5 visits). For predicting the visual acuity, we performed several experiments with different features. First, by considering only previous measured visual acuity, the best accuracy of 0.96 was obtained based on a linear regression. Second, by considering numerical OCT features such as previous thickness and volume values in all retinal zones, the LSTM network reached the highest score ($R^{2}=0.99$). Third, by considering the fundus scan images represented as embeddings obtained from the convolutional autoencoder, the accuracy was increased for all algorithms. The best forecasting results for visual acuity depend on the number of visits and features used for predictions, i.e., 0.99 for LSTM based on three visits (monthly resampled series) based on numerical OCT values, fundus images, and previous visual acuities.

## 1. Introduction

Optical Coherence Tomography (OCT) has become the leading diagnostic tool in modern ophthalmology [1,2], providing various OCT-based biomarkers such as retinal thickness and volume in multiple zones, the presence of intraretinal cystoid fluid, subretinal fluid, alterations of outer retinal layers, and hyperreflective foci. However, this novel tool is not yet able to establish the progression of retinal diseases. Consequently, the ophthalmologist has to analyse the evolution of the condition based on the average thickness and volume on all seven layers and zones of the retina, which is most often a difficult and time-consuming task. Therefore, the human agent is not always able to identify patterns of disease evolution because of the huge number of retinal characteristics (considering all retinal zones, all layers, and the temporal information for each patient visit). Hence, early treatment is not always undergone, resulting in severe vision impairment or even blindness. This is one opportunity for the software agent to complement the ophthalmologist.

Age-related macular degeneration (AMD) is the leading cause of visual impairment and severe visual loss in developed countries. Early and intermediate AMD revolve around the accumulation of drusen and alteration of the retinal pigment epithelium (RPE). Later in the course of the disease, depending on the presence or absence of abnormal new vessels at the level of the RPE, the advanced-stage AMD can be either neovascular (also known as wet) or atrophic (also known as dry). It is at this advanced stage that the central vision starts to decline, gradually in the atrophic type and abruptly in the neovascular type. Despite it already being a major public health problem, the global prevalence of AMD is expected to further rise to 288 million by 2040 [3], heavily increasing the socio-economic burden.

Our task is to forecast the evolution of retinal diseases, to help the ophthalmologist to determine when early treatment is needed in order to prevent significant visual loss.

The acquired data are made up of sequences of visits from multiple patients Age-related Macular Degeneration (AMD) that untreated at the appropriate time, may result in irreversible blindness. Each visit contains OCT B-scans and other retinal features, including visual acuity measurements. Technically, this is a time series forecasting task, where the time series analysis aims to help the ophthalmologist to more quickly understand the factors influencing the disease.

We are looking into two technical challenges. The first concerns how to learn from small-sized time series: there are only short sequences (1–5 visits) of medical observations for each patient. The second is how to reveal the learned model to the human agent, i.e., explainability. Aiming to develop a support system for ophthalmologists to discover trends in retinal diseases, good accuracy might not be enough. Therefore, the outcomes of the machine learning model used should be explainable, meaning that a resembling prediction system as needed must be able to allow the user to understand the decision-making process that led to the result. Besides the confidence, the ophthalmologist could be able to gain more knowledge too. For example, based on the medical condition prognosis and the logics behind the AI model, they would be able to determine whether some parts of the retina have a greater impact on the visual acuity or even whether past treatment had a greater influence.

## 2. The Need for Supporting the Expert’s Decision

Currently, ophthalmologists do not have sufficient data while analysing the OCT B-scans and previous VAs (visual acuity) to predict the evolution of both the retinal structure and vision in patients suffering from late neovascular AMD in order to personalise their treatment plan.

In their patient management, most clinical practices start with an induction phase comprising monthly intravitreal injections for 3 consecutive months. Following this phase, three different regimens have been developed:

1. In pro re nata (PRN), patients are followed each month and treatment is administered when there is presence of disease activity;
2. In treat and extend (T&E), the treatment is administered regardless of disease activity but the intertreatment interval is continuously increased;
3. In a fixed dosing regimen, the treatment is administered at fixed intervals.

Averaging to a bimonthly regimen, as explored by the VIEW study using Aflibercept [4], might appear to be a good middle ground between the aforementioned strategies, but this might lead to over-treating some, with costly outcomes, while under-treating others with loss of vision, thus highlighting the need for a personalized approach—a perfect task for machine learning.

In clinical settings, doctors cannot quantify the amount of intraretinal fluid. They look at the cross-sectional B-scans from a qualitative point of view and then at the ETDRS macular grid to see the difference in volume and thickness from the previous examination. Even though there exist multiple clinical trials evaluating anti-VEGF efficacy, the results differ from those obtained in real-world settings [5] and there is a heterogeneous response between individuals [6] that necessitates tailored personalized treatment schedules.

In the treatment of nAMD, we aim at improving the patient’s VA, given that it is closely connected to their quality of life [7] and their ability to maintain an independent lifestyle. If VA could be predicted using machine learning, we would see three different scenarios:

1. Cases where the VA improves. Having this valuable information and showing it to the patient could increase their willingness to undergo the proposed treatment, their compliance, and their psychological well-being, knowing that a great number of patients fear losing their sight after being diagnosed with exudative AMD [8]. If the prediction could also foresee the best regimen (the number of doses and interval of administration), this could further improve the outcome.
2. Cases where the VA remains unchanged. Knowing that, in its natural history, AMD continues to deteriorate, keeping the current VA would still be considered a successful outcome.
3. Cases where the VA declines. In this scenario, future prediction algorithms should take into account the results for different types of anti-VEGF. We might see different responses depending on the chosen agent and administration regimen, and we could anticipate the ones decreasing the VA. If none of the current clinical agents could improve the patient’s vision, we could guide them to clinical trials testing upcoming new therapies [9]. Another important aspect in this scenario would be to provide adequate mental health support, as recent evidence shows that if implemented at a time during low-vision rehabilitation, this reduces by half the incidence of depressive disorders among nAMD patients [10].

## 3. Preparing the Data

### 3.1. Dataset Description

The dataset was provided by the Department of Ophthalmology of the "Iuliu Hatieganu" University of Medicine and Pharmacy in Cluj-Napoca. It contained 94 patients with age-related macular degeneration (AMD) There were 161 eyes included with more than one medical examination. Each patient had multiple visits, with each one having OCT scans (with XML files that contain retinal thickness and volume features) and the best-corrected visual acuity.

**Figure 1:**
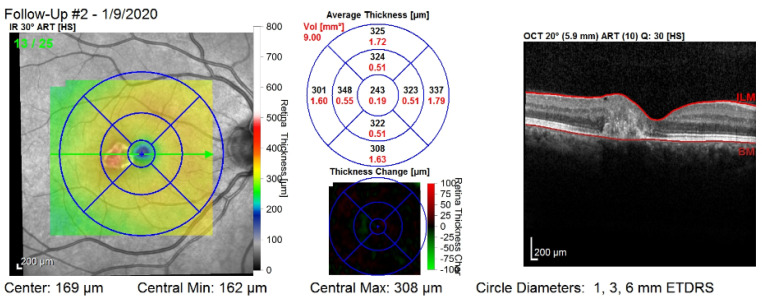
The third visit, i.e., follow-up (#2), of a patient, right eye only. The right column shows that the values are computed on all of the layers of the retina: between the top layer (i.e., ILM) and the bottom layer (i.e., Bruch’s membrane—BM). The middle column shows nine values for the retinal thickness (black color), and nine values for the retinal volume (red color).

Figure 1 shows the third visit of a patient, right eye only. The image corresponds to a single slice from the OCT volume. This slice is the central one illustrated by the green arrow in the left column. Such OCT volumes include 26 slices (or B-scans).

There are some technical challenges that need to be addressed. First, AMD does not necessarily have the same evolution in both eyes, so we can consider having independent sequences for each eye. Second, the sequences of visits have different lengths and the visits take place between different time intervals. Third, the data may be inconsistent between the OCT scans, the retinal features generated by the Heidelberg Spectralis OCT, and the visual acuity values.

### 3.2. Inclusion/Exclusion Criteria

We included patients with late neuvascular AMD who had multiple visits consisting of an initial evaluation and follow-ups. Patients that were in a very advanced stage of the disease or who had surgeries were excluded. We did not include sequences that contained only one visit.

### 3.3. Selecting the Input Features

**Figure 2:**
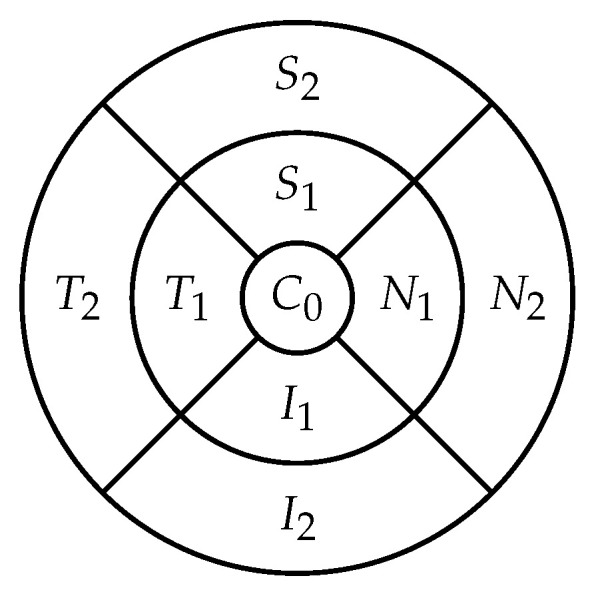
The 9 zones analysed in the retina ($C_{0}$ is the fovea).

The retina is approximately 0.5 mm thick and lines the back of the eye. A circular field of approximately 6 mm around the fovea is considered the centre of the retina, called the macula. Using the Early Treatment Diabetic Retinopathy Study (ETDRS) macular grid within the OCT, we split the retina into 9 zones: $C_{0}$, $N_{1}$, $N_{2}$, $S_{1}$, $S_{2}$, $T_{1}$, $T_{2}$, $I_{1}$, $I_{2}$ (see Figure 2). Retinal direction is indicated by *S* (superior), *I* (inferior), *N* (nasal), and *T* (temporal). The diameters of the three circles are: central subfield (1 mm), inner ring (3 mm), and outer ring (6 mm). In OCT B-scans, the retina layers appear in the right part, as shown in Figure 1.

For each visit, we recorded 3D OCT volumes (with different numbers of OCT B-scans) and fundus scans. For each retinal zone, there were two values: average thickness and volume. As a supplement, there were a central point average thickness value, the minimum and maximum central thickness values, and the total retinal volume. We also had visual acuity measurements for each visit. All of these features were generated by the Spectralis machine from Heidelberg Engineering, except for the visual acuity, which was measured at each visit using Snellen visual acuity charts.

For each medical observation, we had more OCT B-scans, depending on the Spectralis machine’s configuration (FAST, Dense, or Posterior Pole), which determines the number of cross-sectional scans. The machine’s configurations were not uniform, meaning that not all visits had the same number of images, and even so, they might not have been taken in exactly the same position of the eye. This might be an impediment if using the OCT images for prediction.

Moreover, we had an OCT fundus image for each examination and an XML file containing the retinal thickness and volume values in each of the 9 zones from the 3 circle diameters shown in Figure 2. These concentric circle diameters were at 1, 3, and 6 mm from the centre. In the XML file from Listing 1, we had as well the total volume and average thickness of the retina, the minimum and maximum thickness, and the average thickness in the central point.

**Listing 1.** Numerical features of the retina<CentralThickness>0.262</CentralThickness><MinCentralThickness>0.225</MinCentralThickness><MaxCentralThickness>0.333</MaxCentralThickness><TotalVolume>5.664</TotalVolume><Zone>⌴<Name>C0</Name>⌴<AvgThickness>0.259</AvgThickness>⌴<Volume>0.203</Volume>⌴<ValidPixelPercentage>100</ValidPixelPercentage></Zone><Zone>⌴<Name>N1</Name>⌴<AvgThickness>0.299</AvgThickness>⌴<Volume>0.470</Volume>⌴<ValidPixelPercentage>100</ValidPixelPercentage></Zone>

Other relevant numerical data included a variable specifying whether treatment was received or not at the visit, the date of the visit, and the visual acuity value. This visual acuity is our target variable.

### 3.4. The Target Variable—Visual Acuity

The visual function is measured using an ophthalmological examination called the visual acuity test. This test determines the smallest letters that a person can read on a chart, called the Snellen chart. The patient’s visual acuity (VA) is represented by a Snellen (Imperial) fraction based on the following formula: $VA=\frac{Test\;distance}{D}$, where *D* is the distance at which a healthy person can discern the eye chart. Since LogMAR and decimal formats were mostly used, we converted the VA values into the decimal format, since it is the easiest to use and process. In most cases, this implied simply a division, while in other cases, we did not have exact values. Caused by inaccurate measurements, in some cases, we only had a range interval for the visual acuity. For these cases, we used the mean visual acuity value.

The missing visual acuity values were replaced with values that did not influence the sequence for the patient. Inconsistencies between the visual acuity data and OCT data were solved by keeping only data that were common to both of these, or keeping both pieces of data and replacing the missing values.

### 3.5. Normalising the Data

Nonetheless, the other retinal features ranged within different intervals, requiring normalisation, which is an important step for most machine learning algorithms. The min–max normalisation method was used because there were not many outliers and it also does not change the distribution of the data.

### 3.6. Handling the Irregular Time Intervals

An irregular time series is a temporal sequence in which the time intervals between the elements of the sequence are not equal. Similarly, in our data, the medical observations took place at different time intervals for each patient. This could be an issue in the task of predicting the future visual acuity for a patient.

Let the sequence of visits be $v_{1}$, $v_{2}$, …, $v_{n-1}$ and the task to predict the visual acuity for the visit be $v_{n}$. We consider two approaches to predict the visual acuity for the next medical examination of a patient *p* as a function of all past *n* visits:

1. *Time series resampling* to change visit frequency such that they happen at equal time intervals: ${y_{p}}_{n}=f\left({v_{p}}_{1},\ldots {v_{p}}_{n-1}\right)$. After resampling, the missing values should be filled by interpolating the previous and following visits.
2. Consider the sequences of *visits as they occur in reality* and including the timestamps of the visits $t_{i}$ as features, as well as the timestamp $t_{n}$ with which to predict the next visual acuity: ${y_{p}}_{n}=f\left({v_{p}}_{1},\ldots {v_{p}}_{n-1},t_{1},\ldots t_{n}\right)$

Here, ${v_{p}}_{i}={\left[\begin{array}{cccc} {{x_{p}}_{i}}_{1} & {{x_{p}}_{i}}_{2} & \ldots & {{x_{p}}_{i}}_{m} \end{array}\right]}^{⊺}$ is the feature vector for visit *i* of patient *p* containing *m* features ${{x_{p}}_{i}}_{j},\;\text{and}\;j=1,\ldots m$, ${y_{p}}_{n}$ is the visual acuity to be predicted at visit *n* for patient *p*.

### 3.7. Handling the Missing Values

For both previously mentioned cases, the input data can have missing values. Among the most commonly used methods for missing data replacement is linear interpolation, which does not have a significant influence on the dataset. To calculate feature *y* for a visit at time *t*, there must exist two visits at times $t_{1}$ and $t_{2}$, with $t_{1}<t<t_{2}$, and both visits have valid (non-missing) features $y_{1}$ and $y_{2}$.

(1) $$y=y_{1}+\left(t-t_{1}\right)\frac{\left(y_{2}-y_{1}\right)}{\left(t_{2}-t_{1}\right)}$$

This formula can be applied not only on irregular time series (having the timestamps as features), but also on resampled data. Other, more complex methods suggested in the literature include continuous time stochastic models such as GARCH or Kalman filters.

### 3.8. Removing Noise from OCT Scans

Given that some of the OCT images had salt and pepper noise, a median filter was applied. Using a median filter with a kernel of a specific size involves blurring the image by applying the kernel sequentially on it, and each time replacing the pixel from the centre of the kernel with the mean value of all the other pixels in the kernel. More complex deep learning methods for noise reduction consist of different types of CNNs.

### 3.9. Augmentation of Data

Patients did not have the same number of visits, resulting in sequences of different sizes. On the one hand, some clustering and classification methods to determine the evolution of AMD can be used with algorithms suitable for series of unequal length, such as Dynamic Time Warping. On the other hand, for regression techniques, equally sized sequences would be required.

In the dataset, the mean sequence length was 4; thus, it would be a good choice to generate series with at most 4 visits. For example, if a patient has the visits $(v_{1}$, $v_{2}$, $v_{3}$, $v_{4}$, $v_{5})$ and we want to generate a series of 3 visits, the initial sequence can be divided, thus obtaining three series: $(v_{1}$, $v_{2}$, $v_{3})$, $(v_{2}$, $v_{3}$, $v_{4})$, and $(v_{3}$, $v_{4}$, $v_{5})$. The Sliding Window Algorithm is formalised in Algorithm 1.
**Algorithm 1.** Sliding Window Algorithm for time series segmentation.     **Input**: *ts*—multivariate time series of form $(v_{1}$, … $v_{n})$              *k*—the desired size for newly generated series     **Output**: *generated_ts*—vector of all generated series1:**function**SlidingWindow(ts, k)2:    n←lengthoftimeseries3:    generated_ts←newemptyarray4:    **if** k>n **then**5:        **return** generated_ts6:    **else if** k=n **then**7:        generated_ts[0]←ts8:        **return** generated_ts9:    **end if**10:    i←011:    **while** i≤n−k **do**12:        j←013:        new_ts←newemptyarray14:        **while** j<k **do**15:           new_ts[j]←ts[i+j]16:           j←j+117:        **end while**18:        generated_ts[i]←new_ts19:        i←i+120:    **end while**21:    **return** generated_ts22:**end function**

### 3.10. Unsupervised OCT Feature Extraction

Given that we had grayscale images with size 512 × 496, it meant 253,952 pixels (features) for each image. To reduce the features, one can opt for: (i) resizing the images (e.g., to 256 × 256); (ii) extracting the region of interest (ROI); or (iii) applying one of the many algorithms for feature extraction. We opted to apply two algorithms: principal component analysis (PCA) and a convolutional autoencoder.

First, focusing on the statistical variance of the data, PCA is able to determine data components by finding their eigenvectors. The aim is to compute the eigenvectors by maximising the covariance between the features. Consequently, we could use PCA to choose the number of components that are able to capture the maximum amounts of variance from our images. To estimate this, the cumulative explained variance depending on the number of components needed to be determined. The explained variance ratio is represented by the fraction of variance of each component with respect to the total variance of all individual components. Thus, through the cumulative explained variance, we could determine the needed number of components for a specific percentage of variability, preferably the number of components that can describe the overall image set.

**Figure 3:**
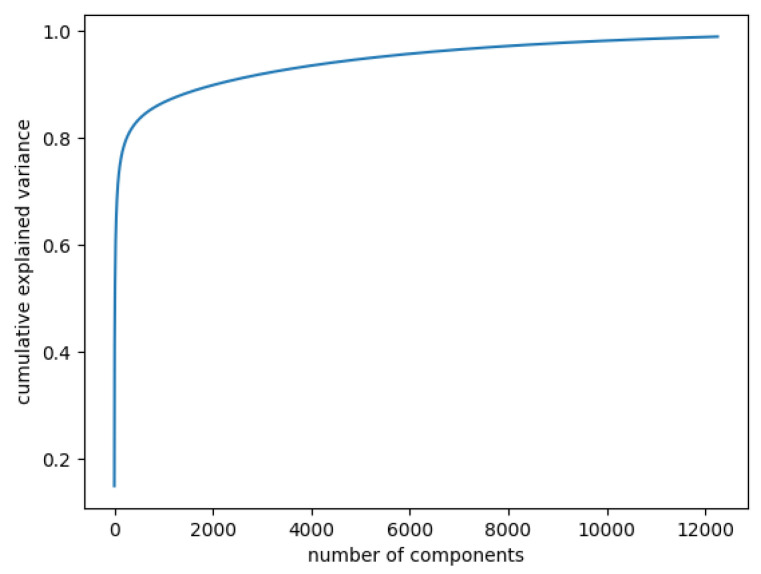
Cumulative explained variance: 99% of the data are represented with 12,000 components.

**Figure 4:**
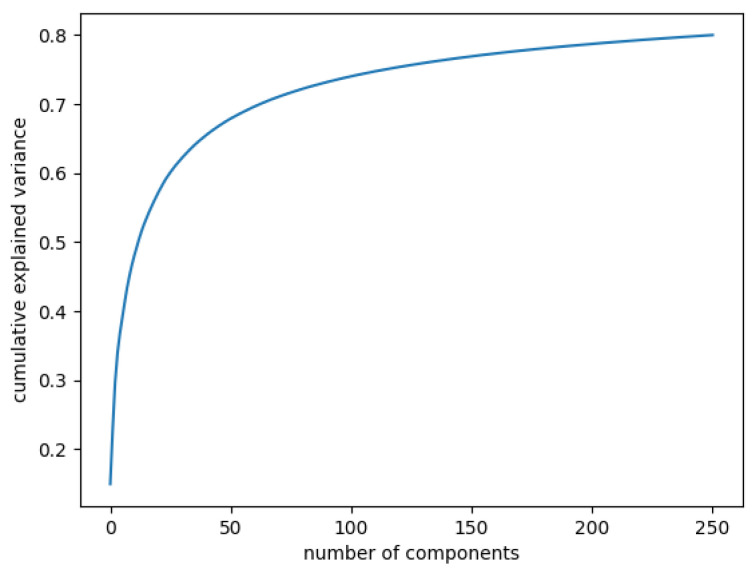
Cumulative explained variance: 80% of the data are represented with 250 components.

In Figure 3, the curve quantifies how much of the total variance in approximately 12,000 dimensions is contained within the first *n* components. Here, 99% of the images can be represented using 12,000 components, but 12,000 is still too large for the number of features. Being able to represent 80% of the data would also be a good percentage; Figure 4 shows that, in this case, we would need slightly more than 250 components.

Second, autoencoders are a type of artificial neural network that have two major components: an encoder and a decoder. The encoder is used to generate a latent space representation as an embedding of the input, and the decoder learns to reconstruct the input based on the code.

An explainable method would be to make use of a convolutional autoencoder (CAE), which applies multiple convolutions on the images in the decoder and then learns to reconstruct them. Several studies [11,12] have shown that CAEs can obtain better results in medical imaging, focusing more on the relevant features, through a more natural decomposition in the latent space. It was also shown that, regarding choosing the code size of an autoencoder, the rules commonly used in PCA can also be applied to autoencoders.

Thereby, a convolutional autoencoder with the latent code size of 256 would be a good choice. The encoder performs multiple convolutions on the images, returning a vector of length 256, while the decoder learns to reconstruct the OCT input images during the training, updating all the weights.

Autoencoders were also used in the pipeline from [13], firstly to encode the cross-sectional OCT scans and then to encode the obtained representation. Even though such a method could improve our results, the approach would not be exactly suitable because of our data inconsistencies. Each patient visit had a different number of OCT scans, depending on the mode used by the Spectralis system. Some research papers, however, try to predict disease evolution from OCT fundus scans. Using only the OCT fundus scans could be more manageable.

### 3.11. Selecting Numerical Features

Based on the existing research, some retinal layers and zones may have a greater influence on the visual function of people with AMD than others. However, we only had the numerical features of the retinal zones, while most studies use features specific to the retinal layers. Despite this, some zones have been shown to be more influential with respect to the visual function, i.e., zones such as the fovea or the closest ones to the fovea, so domain knowledge can be also used for feature selection. The current focus was to determine the zones that are most correlated to the visual acuity evolution, from a statistical point of view. We used two approaches, detailed below.

#### 3.11.1. Univariate Analysis

In order to select which of these to use first of all, an analysis concerning the relationship between the retinal features and the visual acuity was performed. The correlation between series of features can be determined through correlation coefficients such as Pearson or Spearman. Both of these coefficient values can range between −1 and 1, values near 0 revealing no correlation or a weak correlation, while values closer to the endpoints of this interval suggest a stronger correlation. The difference between the Pearson coefficient (*r*) and Spearman’s (ρ) is that the Pearson coefficient evaluates a linear correlation, while Spearman’s evaluates only a monotonic correlation. These coefficients were computed for two features *x* and *y* using Equations (2) and (3).

(2) $$r=\frac{\sum_{i=1}^{n}\left(x_{i}-\bar{x}\right)\left(y_{i}-\bar{y}\right)}{\sqrt{\sum_{i=1}^{n}{\left(x_{i}-\bar{x}\right)}^{2}\sum_{i=1}^{n}{\left(y_{i}-\bar{y}\right)}^{2}}}$$

(3) $$\rho =1-\frac{6\sum d_{i}^{2}}{n(n^{2}-1)}$$

where *n* is the number of observations for the features and *d* is the difference between the two ranks of each observation. Based on Equations (2) and (3), we could determine the retinal attributes that had a greater effect on the change in the visual acuity. The results in Table 1 show that even if there is not any strong correlation, the zones most correlated with the visual acuity are $C_{0}$ (the fovea) and $N_{1}$ (inner nasal zone). To investigate whether these features together had a higher impact, a multivariate analysis was required.

#### 3.11.2. Multivariate Analysis

Besides using domain knowledge, there are several major ways to perform multivariate feature selection, including: (i) using a filter, (ii) using a wrapper, and (iii) embedded methods [14].

First, the filter methods are used in the preprocessing pipeline, and they involve choosing the best features based on a specific ranking. For instance, the previous correlation coefficients can be applied, afterwards selecting the most strongly correlated values among them. The idea would be to choose more strongly correlated features, but, in our case, we did not have very strong correlations (see Table 1). Another filter method would be to not use the variables that have a variance of 0, which denotes that they do not change in time. Such features can even worsen our models. However, having multiple time series in this case, the variance of the feature must be 0 for all the series so as to remove it, which is highly improbable.

**Figure 5:**
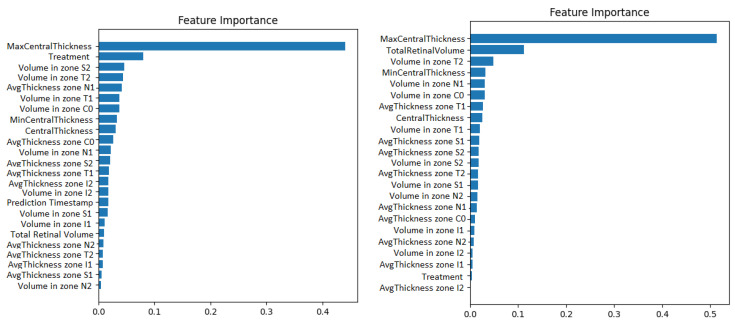
Gradient boosting machine showing how much the numerical OCT features influence the prediction of visual acuity. The case with prediction timestep as a features appears on the left. The case with 1-month resampling appears on the right.

Second, the wrapper methods use greedy algorithms to select the features that obtain the best results against a specific learning algorithm. One disadvantage could be that these methods are computationally expensive. An example of such a method is the Recursive Feature Elimination algorithm (RFE). It recursively removes features and retrains the chosen model, until the best performance is obtained. Along with the best performance, the method is interpretable too because it chooses the features based on the feature importance weight’s computation. The Recursive Feature Elimination technique with cross-validation was applied using the implementation from the scikit-learn library. The model used was a gradient boosted machine. The feature importance obtained for both the original data and for the resampled time series can be visualized in Figure 5.

Third, embedded methods use learning algorithms that have their own feature selection methods built in. Some examples are lasso regularization in linear regression and random forest regression. These are machine learning algorithms typically used for regression, furthermore quantifying feature importance. Thus, they can be used for feature selection and even for post-hoc explainability after predictions.

We used the lasso regression implementation from scikit-learn. Cross-validation was applied when training the estimators, in order to obtain the best $R^{2}$ score. The features having weights equal to 0 were removed.

## 4. Running Experiments

### 4.1. Visual Acuity Forecasting

For predicting future visual acuities, we used multiple regression algorithms. Besides the classical linear regression, the focus was placed more on deep neural networks and ensembles, due to their greater performance. Gradient boosting, random forest, and extremely randomised trees regression algorithms were experimented with K-fold cross-validation, in order to obtain a more accurate evaluation of the algorithm’s performance.

**Figure 6:**
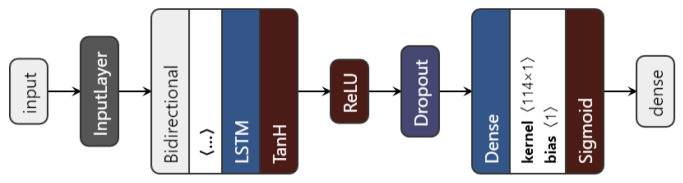
The proposed LSTM architecture.

We introduced a bidirectional wrapper over the recurrent layer, making the network a bidirectional recurrent neural network. Bidirectional RNNs are useful, especially in time series, since they are able to learn patterns from temporal sequences in both directions, thus helping in the prediction process. Moreover, the custom model can use any of the three previously mentioned RNN units. The proposed architecture is shown in Figure 6.

The data to be fed to the network consist of the augmented time series *〈no_series, timesteps, no_features〉*. Then, depending on the chosen type of network (based on the *nn_type* variable), the inputs go through the chosen recurrent layer. The LSTM layer uses an L1 regulariser with α=0.01 to regularise the input feature weights. As a regularisation method, and also to avoid overfitting, a dropout layer is used. Finally, the dense layer with one unit gives the predicted visual acuity. The sigmoid activation function is applied here in order to restrict the interval range of the visual acuity outcome, to be between 0 and 1.

The neural network models are trained using early stopping, meaning that there is not a fixed number of epochs, and the best-performing model is always saved. The loss is computed using Mean Squared Error (MSE), but other metrics are used as well: MAE, RMSE, RMSPE, or R${}^{2}$.

### 4.2. Experimental Setup

The data consist of irregular time series of medical examinations, with varying lengths, having as variables: (i) the target—visual acuity values; (ii) the features—18 numerical OCT variables; (iii) the OCT images.

To obtain the image embeddings, a deep unsupervised dimensionality reduction method was used, specifically a convolutional autoencoder. All 14,337 OCT images were split into three subsets for training (60%), validation (20%), and testing (20%).

For clustering, we were able to use the original sequences, disregarding their irregularity due to the Dynamic Time Warping metric.

For time series classification and forecasting, the original 115 time series were preprocessed and augmented. Two main approaches were considered. First, the time series were resampled using interpolation, such that all time intervals were equal to 1 month. Second, we simply used the time interval values as features, represented as the number of months from the first visit in each sequence. The series were then segmented in order to obtain more series of the same size. Hence, time series of size 2, 3, and 4 were generated (see Table 2).

After augmentation, the data were also split into 3 parts: training (60%), validation (30%), and testing (10%) data. Only 10% of the data were kept for testing, given that we had a small dataset. This applied only to the case of deep neural networks. For shallow machine learning algorithms, 10-fold cross validation was performed in all cases, having only train–test splits.

## 5. Results

### 5.1. Convolutional Autoencoder

**Figure 7:**
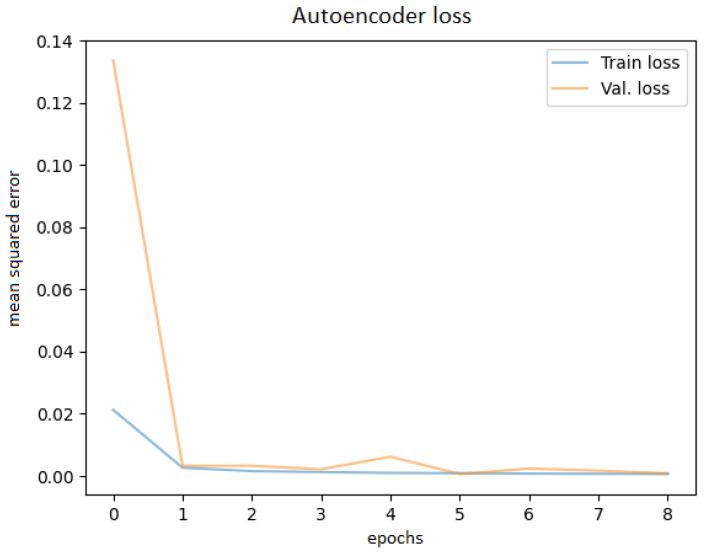
Autoencoder training and validation plot.

After training the autoencoder with different hyperparameters, always saving the best model and using early stopping to avoid overfitting, the best performance was obtained. Various latent code sizes were tested, even though the most acceptable size would be 256 according to the principal component analysis. Tests were attempted to determine whether training with preprocessed techniques (noise filtering, cropping the ROI) would bring better results, but they did not; thus, the original images were used. Before feeding the OCT images into the network, they were resized to 256 × 256 pixels. The best learning curve is shown in Figure 7.

**Figure 8:**
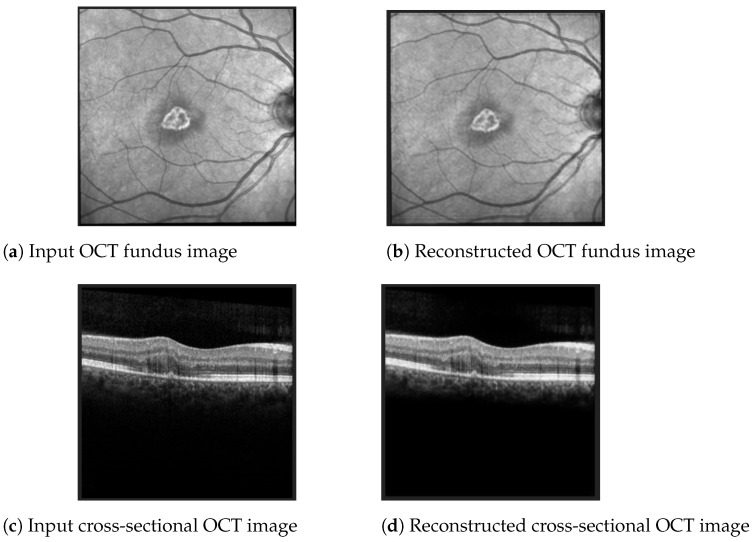
Examples of OCT scan reconstruction and noise filtering using the convolutional autoencoder.

The best Mean Squared Error (MSE) obtained on the test data was 0.0007, meaning that the model was able to reconstruct the OCT images from the embeddings almost perfectly. Examples of OCT image reconstructions are depicted in Figure 8, showing that this autoencoder can also be used for noise reduction. The model was able to reconstruct the OCT images from the latent embeddings. Noticeably, the reconstruction worked very well, emphasising the most important retina features and even filtering out some noise.

### 5.2. Predicting Disease Evolution: Better or Worse

First, clustering of the original time series, with varying sizes and all available features, was carried out in order to determine two clusters of disease evolution: amelioration or worsening. K-means with the Dynamic Time Warping metric resulted in the best accuracy of 56% regarding the visual acuity evolution. Hence, clustering might not be the most appropriate for this task, or other data should be used.

**Figure 9:**
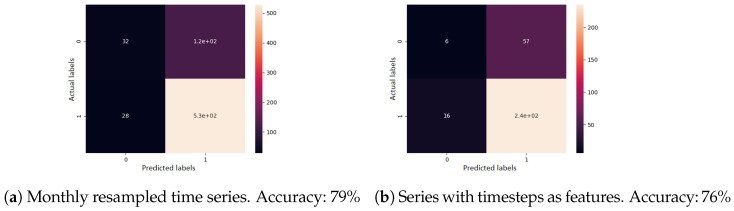
Confusion matrix results for the disease evolution classification using gradient boosting classifier. Label 0 represents a good disease evolution, while 1 stands for a poor evolution. The matrix column represents the actual label.

Second, for supervised learning, a gradient boosting classifier was used. A single previous visit was provided as input, the task being to determine the evolution type. All features were used here as well. The confusion matrix of the cross-validated predictions, for each sequence modelling approach, can be seen in Figure 9. When visits were resampled to one month each, the accuracy was 79%. When visits were given as irregular time series, the accuracy was 76%.

### 5.3. Visual Acuity Forecasting

Experiments for predicting the future visual acuity of patients with AMD were achieved using multiple machine learning algorithms. The input data used were time series of patient visits containing the following types of features:

- The previous visual acuity values together with information regarding when treatment was received by each patient (Table 3);
- The numerical OCT features (thickness, volume values in all retinal zones) (Table 4);
- The OCT fundus scan images represented as embeddings, obtained from the convolutional autoencoder (Table 5).

The best overall results were achieved using only past visual acuity data (including when treatments were received), obviously showing that any future visual acuity value for patients with AMD is mostly influenced by the past visual acuities observed. Shallow machine learning algorithms performed surprisingly well in this case, even better than more advanced methods such as deep neural networks. The best results in all cases were achieved mainly with linear regression. All algorithms were validated using 10-fold cross-validation. The performance was evaluated using the $R^{2}$ metric (see Table 6).

**Figure 10:**
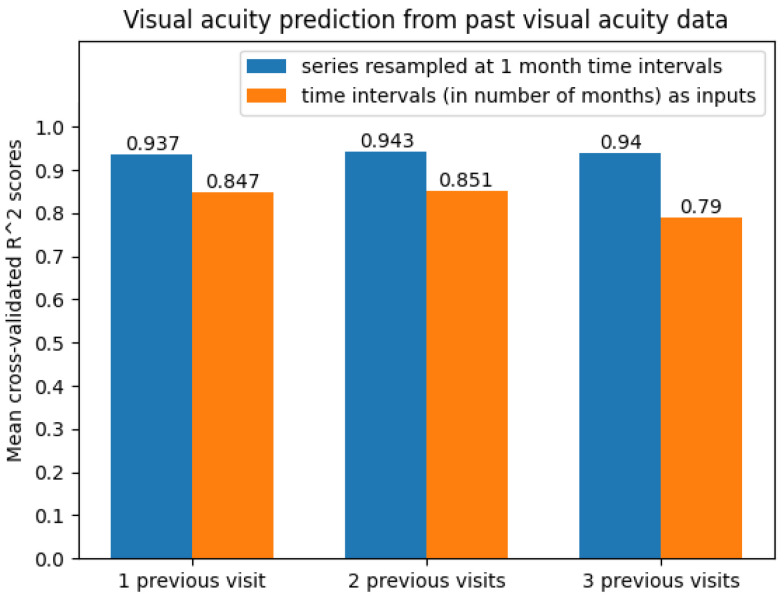
Average cross-validated $R^{2}$ scores when predicting future visual acuity only from previous visual acuity data.

The best results were obtained on the time series resampled at 1-month time intervals. The average scores can be seen in Figure 10. Clearly, the predictive models were more accurate when performing 1-month time series resampling as a preprocessing step.

Experiments for predicting future visual acuity based on the numerical OCT data (22 features) and the feature that indicated whether treatment was received at a given visit were performed as well. The performance of the learning algorithms was evaluated using the MSE, MAE, RMSE, R${}^{2}$, and RMSPE metrics. Machine learning performed poorly in this scenario, with deep learning showing significantly better results. Hence, only the neural network model results were taken into consideration. The best results for each time series size (i.e., number of previous visits) and each model are shown in Table 7, Table 8 and Table 9, respectively. They suggest the same as in the previous experiments, namely that the best performance was achieved by resampled time series.

**Figure 11:**
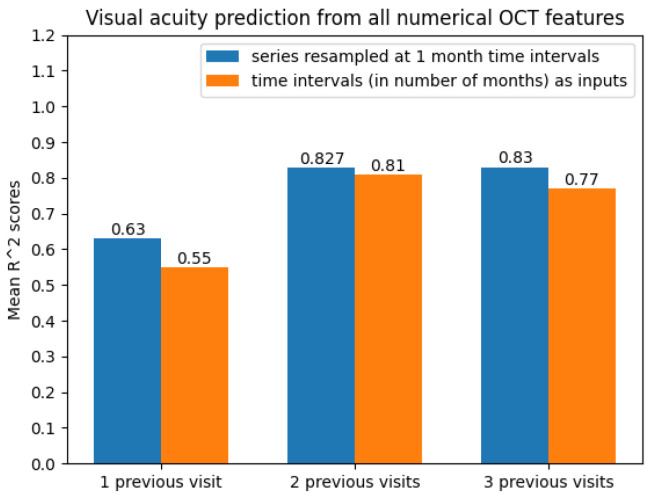
Average $R^{2}$ scores when predicting future visual acuity from all numerical OCT data.

With regard to the modelling approach, time series with two and three previous visits showed promising prediction results. This can also be observed from Figure 11.

**Figure 12:**
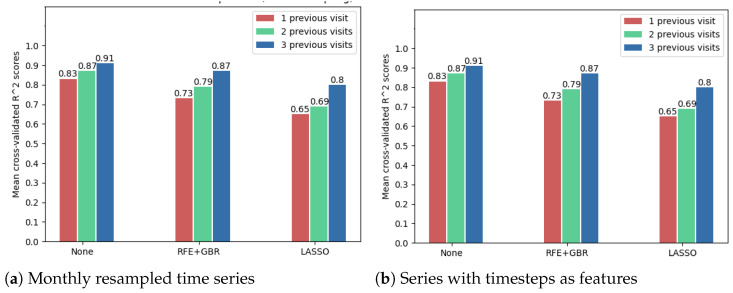
Comparison of feature selection methods’ performance when predicting future visual acuity from all OCT data. Average cross-validated R${}^{2}$ scores for all neural networks were computed.

The following results show that using the OCT fundus image embeddings as inputs (256 new features), in addition to the numerical OCT features, may improve the forecasting results. Because of the large number of features in this case, the feature selection techniques were compared to decide whether such a technique is needed. However, Figure 12 shows that the best model accuracies were obtained without further applying a feature selection method. Noticeably, from these cross-validated accuracies, one can once again arrive at the conclusion that time series resampling improves the forecasting results.

Consequently, all features (23 numerical; 1 describing weather treatment was received; 256 features from the OCT fundus images) were used without any additional feature selection technique. The best model results for each number of previous visits (1, 2, 3) are shown in Table 10, Table 11 and Table 12.

**Figure 13:**
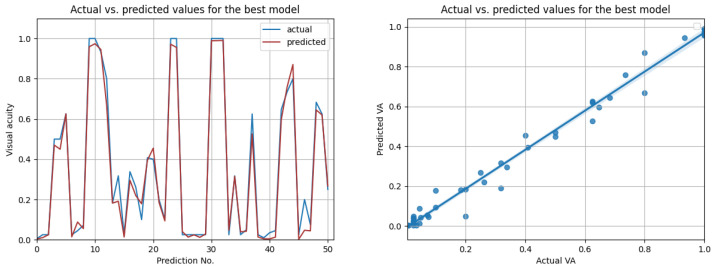
Actual vs. predicted VA values for the best model (LSTM with $R^{2}=0.98$, for three previous visits). The second figure shows that the actual and predicted visual acuities are highly correlated (r=0.993).

The most promising results were again achieved in the case of resampled series. As shown in Table 12, the highest overall $R^{2}$ score of 0.98 was achieved by the best LSTM model, when using input data from the prior three medical observations. Figure 13 compares the actual visual acuities in the test data, compared to the predicted ones. Clearly, the prediction errors are significantly low. The right-side image from Figure 13 shows the correlation between the actual and predicted values. With a Pearson correlation coefficient of 0.993, a high correlation is implied.

#### Best Overall Forecasting Results

Based on the previous experiments, the best final performance results (R${}^{2}$ scores) are summed up in Table 13, together with the best models corresponding to them. Moreover, so as to improve the recurrent neural network predictions, the past visual acuities were used in addition to the OCT features and the features concerning treatments. Hence, one more line was added to the table, showing the best model results when using all features as input.

**Figure 14:**
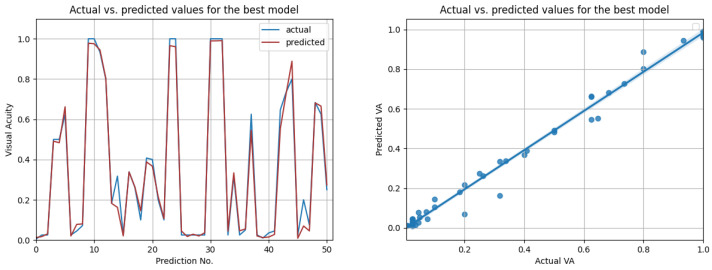
Actual vs. predicted VA values for the best model using all data (LSTM with $R^{2}=0.99$, for three previous visits). The second figure shows that the actual and predicted visual acuities are highly correlated (r=0.994).

**Figure 15:**
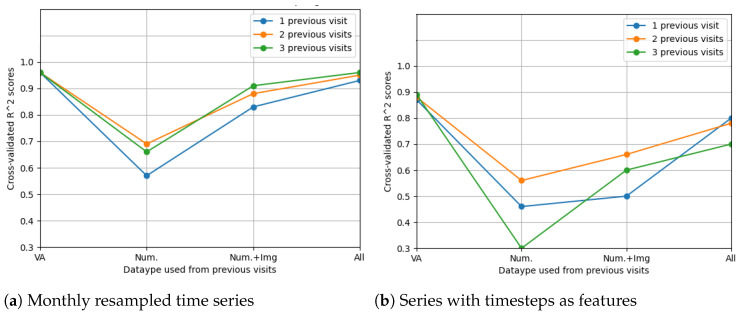
Comparison of feature selection methods’ performance when predicting future visual acuities from all OCT data. Average cross-validated $R^{2}$ scores for all neural networks were computed.

The LSTM network reached the highest score ($R^{2}=0.99$) in the resampling scenario, considering the situation with all features from three previous visits. The other metrics (MSE=0.0016;MAE=0.024;RMSE=0.04;RMSPE=0.0038) indeed suggest high prediction accuracy, even higher than in the case when only OCT data were used. In Figure 14, the prediction results on the test data are compared to the actual results. Figure 15 shows the cross-validated LSTM scores with resampling (left) and without resampling (right).

## 6. Related Work

In analysing the evolution of retinal diseases, several studies have attempted to find the relationship between the structural changes in the OCT biomarkers and the visual acuity. From a technical point of view, regression is usually used to demonstrate that there is a correlation between retinal characteristics from OCT scans and the best-corrected visual acuity. We emphasise how machine learning algorithms are applied for this, as well for predicting future visual acuities from time series of medical observations.

### 6.1. Machine Learning for Analysing the Relationship between OCT and Visual Acuity

Pelosini et al. [15] have used linear regression to demonstrate that the volume of retinal tissue between the plexiform layers predicts 80.7% of visual acuity. Their study included 129 eyes from 81 patients with macular oedema. The analysed data contained the best-corrected visual acuity values and spectral domain OCT scans for each patient. Each OCT fundus scan was divided into five concentric radii (concentric rings of 500, 1000, 1500, 2000, and 2500 μm from the fovea). The stepwise linear regression model developed determined that the visual acuity can be predicted from the tissue volume between the inner and outer plexiform layers, in the rings up to 1000 μm. Conversely, the central macular thickness up to 1000 μm from the foveal centre was found not to be a strong predictor of the visual acuity. Similarly, using regression, Ting et al. [16] have found a significant correlation between the foveal thickness and the visual acuity (P=0.02) of patients with age-related macular degeneration. Rohm et al. [17] have found as well that the foveal volume and total volume have a strong impact on future visual acuity, besides the prior visual acuity.

The evolution of AMD has been analysed using regression [18]. Time series data with time intervals of 3 months, from 38 patients (61 eyes) with intermediate AMD, were used to predict the drusen progression, which mostly influences the disease. Segmentation of the drusen was performed for the determination of some characterising features such as the drusen thickness, the outer nuclear layer (ONL) thickness, and the total hyper-reflective foci (HRF) volume in the retina. Based on this automated image analysis method, Bogunovic et al. have developed a model to predict the drusen progression within the next 2 years, obtaining an AUC of 0.75.

Bogunovic et al. have attempted to predict the visual acuity of patients with AMD during their treatment [19]. The medical observations of 32 subjects took place at 2-week time intervals. Relevant features were extracted from the OCT scans, from all nine zones of the retina, through an Early Treatment of Diabetic Retinopathy (ETDRS) grid centred on the fovea. Random forest regression was used to predict the VA of the patients, two visits after their treatment induction phase, meaning one month later. Bogunovic et al. determined that the most important OCT biomarkers were the mean ONL thickness in the inferior parafoveal region at the second visit and the intraretinal fluid area in the superior parafoveal region at the sixth visit. The correlation coefficient between the measured VA and the predicted one was r=0.57.

Furthermore, the occurrence of intraretinal fluid corresponds to lower visual acuity values in many studies. Ting et al. [16] obtained P<0.001, denoting a highly significant relation between the presence of macular oedema and decreased visual acuity, from a statistical point of view. In another study, Seebock et al. performed segmentation of the areas that contained intraretinal cystoid fluid using an anomaly detection approach [20]. The proposed method trains a Bayesian U-Net on healthy data, using weak labels of the retinal layers generated with the graph-based segmentation method, as detailed by Garvin [21]. This approach could also be helpful for determining relevant OCT biomarkers.

Additionally to biomarkers such as retinal thickness and volume in different retinal zones and layers, drusen, or intraretinal fluid, OCT images can be used to forecast best-corrected visual acuity values. A comparative performance analysis was carried out between four predictive models in [22]. The dataset contained OCT biomarkers and fundus scan images from 140 patients with AMD. The medical examinations of the patients took place at 6-month intervals. The first predictive model used only the OCT fundus images as inputs, the second and third used different OCT biomarkers, while the fourth model used both data types. Evaluating the models, the first one achieved an AUC=0.80, the second and third an AUC=0.82, and the last model an AUC=0.85, showing that the fundus images together with the OCT biomarkers may become more relevant in predicting retinal disease evolution.

OCT cross-sectional scans could be employed to extract structural OCT features that may predict future visual acuities. Besides identifying retinal biomarkers such as the ones previously mentioned, feature extraction methods can be used. An unsupervised biomarker extraction method from 3D OCT volumes has been proposed by Waldstein [13]. The solution comprises a machine learning pipeline that consists of two autoencoders. The first autoencoder extracts the local features of each OCT B-scan in the volume, and then the second one compresses the local features into the final global features of the volume. After obtaining these new OCT features, an *r* correlation of up to 0.73 was obtained with regard to the visual acuity. Autoencoders are widely used in existing research [23,24] to extract relevant features from large medical images, not only in ophthalmology.

### 6.2. Time Series Forecasting

Predicting the evolution of retinal diseases given temporal sequences of medical observations reduces to a time series forecasting task. Machine learning techniques such as linear regression and random forest have already been used; however, there is a large scale of suitable algorithms for regression, including deep learning algorithms.

Rohm et al. [17] have compared five machine learning algorithms (i.e., AdaBoost, gradient boosting, random forest, extremely randomised trees, and lasso regression) for predicting best-corrected VA for patients with AMD. The aim was for a 3-month and 12-month prediction. The input data were retinal thickness and macular volumes from all nine zones of the retina (obtained from the Spectralis Heidelberg Eye Explorer software), from the previous one, two, three, and four visits of each patient. Overall, based on the MAE and RMSE scores and using 10-fold cross validation, the best-performing algorithm was lasso linear regression. The best results were obtained for the shortest-term prediction (3 months), for two previous visits (lasso results in this case: RMSE=0.14, MAE=0.11).

Banerjee et al. [25] have evaluated the efficiency of a deep learning model (RNN), compared to random forest, for predicting the risk of AMD progression from OCT scans. Forecasting from time series of observations (between 1 and 15 past medical observations), their [26] Long Short-Term Memory-based recurrent neural network displayed significantly greater performance. The proposed deep learning model achieved an AUC-ROC of up to 0.96 using 10-fold cross validation.

RNN architectures are known to be the most suitable for time series prediction, depending as well on the number of parameters and the size of the sequences. Due to the common issues with exploding and vanishing gradients, LSTM [26] and Gated Reccurrent Unit [27] (GRU) networks may be used. According to Lim and Zohren [28], attention mechanisms together with RNNs usually lead to improvements when learning long-term dependencies. Such transformer architectures are suitable for time series [29]. However, in this context, learning from small time series should not necessarily require attention mechanisms.

Apart from RNNs, convolutional neural networks (CNN) can be adapted for time series, especially when using OCT images for prediction. For instance, CNNs have been used to predict the best-corrected VA of patients with neovascular AMD, from OCT scans [30]. Transfer learning with ResNet50 has been used to predict whether the future visual acuity (1 month and 12 months later) is below or above some thresholds: 20/40, 20/60, and 20/200 (in Snellen imperial representation) [31]. The 1-month forecasting results were $R^{2}=0.67$ and RMSE=8.6 for the studied eyes, respectively, and $R^{2}=0.84$ and RMSE=9.01 for the fellow eyes. For a 12-month prediction, results were $R^{2}=0.33$ and RMSE=14.16 for the studied eyes, respectively, $R^{2}=0.75$ and RMSE=11.27.

The classification method from past OCT image series proposed by Romo-Bucheli et al. [32] aims to forecast the need for treatment. Based on the prior OCT scans from the last three patient visits, the CNN predicts whether there is a high, intermediate, or low treatment requirement. The proposed architecture integrates DenseNet [33], which extracts features from multi-tile OCT B-scans, with an RNN that learns from time dependencies. The highest accuracy (0.9) was obtained for predicting a high need for treatment.

## 7. Conclusions

We targeted the progression of age-related macular degeneration by means of machine learning technologies, aiming at helping the ophthalmologist to determine when early treatment is needed. We collected a dataset containing 94 patients with AMD and there were 161 eyes included with more than one medical examination. We applied linear regression, gradient boosting, random forest and extremely randomised trees, a bidirectional recurrent neural network, an LSTM network, and a GRU network to handle technical challenges such as learning from small time series, handling different time intervals between visits, or learning from different numbers of visits for each patient (1–5 visits). For predicting the visual acuity, we conducted several experiments with different features. First, by considering only the previously measured visual acuity, the best accuracy of 0.96 was obtained based on a linear regression. Second, by considering numerical OCT features such as previous thickness and volume values in all retinal zones, the LSTM network reached the highest score ($R^{2}=0.99$). Third, by considering the fundus scan images represented as embeddings obtained from the convolutional autoencoder, the accuracy was increased for all algorithms. The best forecasting results for visual acuity depend on the number of visits and features used for predictions, i.e., 0.99 for LSTM based on three visits (monthly resampled series) based on numerical OCT values, fundus images, and previous visual acuities. Shallow machine learning algorithms performed surprisingly well in this case, even better than more advanced methods such as deep neural networks.

## Figures and Tables

**Table 1:** Univariate correlation of OCT features with visual acuity.

OCT Feature	Zone	Pearson Coefficient *r*	Spearman Coefficient ρ
Average thickness	$C_{0}$	−0.19	−0.21
Average thickness	$N_{1}$	−0.22	−0.22
Average thickness	$N_{2}$	0.01	−0.02
Average thickness	$S_{1}$	−0.14	−0.15
Average thickness	$S_{2}$	−0.11	−0.12
Average thickness	$T_{1}$	−0.15	−0.14
Average thickness	$T_{2}$	0	0.01
Average thickness	$I_{1}$	−0.12	−0.12
Average thickness	$I_{2}$	−0.09	−0.08
Volume	$C_{0}$	−0.21	−0.23
Volume	$N_{1}$	−0.22	−0.22
Volume	$N_{2}$	0.01	0.02
Volume	$S_{1}$	−0.13	−0.13
Volume	$S_{2}$	−0.11	−0.13
Volume	$T_{1}$	−0.15	−0.16
Volume	$T_{2}$	0	0
Volume	$I_{1}$	−0.12	−0.11
Volume	$I_{2}$	−0.1	−0.1
Central average thickness	-	−0.19	−0.19
Minimum central thickness	-	−0.17	−0.18
Maximum central thickness	-	−0.13	−0.16
Total volume	-	−0.13	−0.12

**Table 2:** Number of augmented time series depending on the sequential modelling approach and the size of each series.

Method	Time Series Size		
	2	3	4
Monthly resampled series	712	597	509
Timesteps as features	314	199	126

**Table 3:** ${Experiment}_{1}$: Forecasting VA${}_{t+1}$ from previous VA data.

Aim:	Predicting visual acuity based on previous VA${}_{i}$ measurements available
Time series:	Both monthly resampled visits and irregular visits (with timesteps included as features).
Algorithms:	Linear regression, gradient boosting regression, random forest regression, extremely randomised trees, simple RNN, LSTM network, GRU network
Results:	Best accuracy 0.96—linear regression on monthly resampled series (Table 6)

**Table 4:** $Experiment_{2}$: Forecasting VA${}_{t+1}$ from the numerical OCT features.

Aim:	Predicting visual acuity based on previous thickness and volume values
Time series:	Both monthly resampled visits and irregular visits (with timesteps included as features).
Visits:	1 visit, 2 visits, and also 3 visits
Algorithms:	Simple RNN, LSTM network, GRU network
Results:	Table 7, Table 8 and Table 9

**Table 5:** $Experiment_{3}$: Forecasting VA${}_{t+1}$ from the numerical OCT features and fundus images.

Aim:	Predicting visual acuity based on previous thickness and volume values in all retina zones plus fundus image embeddings as 256 new features
Time series:	Both monthly resampled visits and irregular visits (with timesteps included as features).
Algorithms:	Simple RNN, LSTM network, GRU network
Results:	Table 10, Table 11 and Table 12

**Table 6:** $R^{2}$ results when forecasting from past visual acuity data (including information on when treatment was received).

Algorithm	Monthly Resampled Series			Timesteps as Features		
	Visits = 1	Visits = 2	Visits = 3	Visits = 1	Visits = 2	Visits = 3
Linear Regression	**0.96**	**0.96**	**0.96**	**0.87**	**0.88**	**0.89**
Gradient Boosting Regression	0.95	**0.96**	0.94	0.85	0.86	0.84
Random Forest Regression	0.95	0.93	0.94	0.85	0.86	0.86
Extremely Randomised Trees	0.95	0.91	0.94	0.83	0.83	0.85
Simple RNN Network	0.92	0.95	0.93	0.85	0.84	0.69
LSTM Network	0.91	0.94	0.93	0.84	0.84	0.70
GRU Network	0.92	0.95	0.94	0.84	0.85	0.72

**Table 7:** Results when forecasting from all numerical OCT features, from one previous visit.

Algorithm	Monthly Resampled Time Series					Timesteps as Input Features				
	MSE	MAE	RMSE	$R^{2}$	RMSPE	MSE	MAE	RMSE	$R^{2}$	RMSPE
Simple RNN Network	0.03	0.13	0.22	0.64	0.03	0.04	0.14	0.21	0.55	0.04
LSTM Network	0.04	0.13	0.17	0.65	0.03	0.04	0.16	0.21	0.56	0.03
GRU Network	0.04	0.15	0.23	0.59	0.03	0.05	0.15	0.21	0.53	0.04

**Table 8:** Results when forecasting from all numerical OCT features, from two previous visits.

Algorithm	Monthly Resampled Time Series					Timesteps as Input Features				
	MSE	MAE	RMSE	$R^{2}$	RMSPE	MSE	MAE	RMSE	$R^{2}$	RMSPE
Simple RNN network	0.03	0.09	0.18	0.83	0.02	0.02	0.13	0.15	0.81	0.04
LSTM Network	0.02	0.09	0.13	0.83	0.02	0.02	0.12	0.14	0.83	0.03
GRU Network	0.02	0.09	0.14	0.82	0.01	0.03	0.13	0.16	0.80	0.04

**Table 9:** Results when forecasting from all numerical OCT features, from three previous visits.

Algorithm	Monthly Resampled Time Series					Timesteps as Input Features				
	MSE	MAE	RMSE	$R^{2}$	RMSPE	MSE	MAE	RMSE	$R^{2}$	RMSPE
Simple RNN Network	0.02	0.08	0.14	0.81	0.03	0.02	0.12	0.15	0.80	0.05
LSTM Network	0.02	0.08	0.13	0.85	0.02	0.03	0.13	0.17	0.76	0.06
GRU Network	0.02	0.07	0.13	0.84	0.03	0.03	0.13	0.18	0.75	0.06

**Table 10:** Results when forecasting from all OCT data (including fundus image embeddings), from one previous visit.

Algorithm	Monthly Resampled Time Series					Timesteps as Input Features				
	MSE	MAE	RMSE	$R^{2}$	RMSPE	MSE	MAE	RMSE	$R^{2}$	RMSPE
Simple RNN Network	0.02	0.05	0.09	0.94	0.04	0.02	0.11	0.15	0.79	0.01
LSTM Network	0.01	0.05	0.07	0.95	0.03	0.02	0.11	0.14	0.80	0.01
GRU Network	0.02	0.06	0.08	0.95	0.03	0.02	0.11	0.14	0.81	0.01

**Table 11:** Results when forecasting from all OCT data (including fundus image embeddings), from two previous visits.

Algorithm	Monthly Resampled Time Series					Timesteps as Input Features				
	MSE	MAE	RMSE	$R^{2}$	RMSPE	MSE	MAE	RMSE	$R^{2}$	RMSPE
Simple RNN Network	0.01	0.06	0.07	0.95	0.02	0.02	0.09	0.14	0.83	0.01
LSTM Network	0.01	0.05	0.07	0.95	0.01	0.02	0.09	0.14	0.87	0.01
GRU Network	0.01	0.05	0.08	0.95	0.01	0.03	0.12	0.16	0.77	0.03

**Table 12:** Results when forecasting from all OCT data (including fundus image embeddings), from three previous visits.

Algorithm	Monthly Resampled Time Series					Timesteps as Input Features				
	MSE	MAE	RMSE	$R^{2}$	RMSPE	MSE	MAE	RMSE	$R^{2}$	RMSPE
Simple RNN Network	0.01	0.06	0.08	0.95	0.01	0.03	0.13	0.17	0.77	0.05
LSTM Network	0.002	0.03	0.05	0.98	0.004	0.02	0.11	0.15	0.81	0.04
GRU Network	0.01	0.05	0.08	0.95	0.01	0.03	0.12	0.17	0.77	0.04

**Table 13:** Best forecasting results depending on the input data.

Algorithm	Monthly Resampled Series			Timesteps as Features		
	Visits = 1	Visits = 2	Visits = 3	Visits = 1	Visits = 2	Visits = 3
VA	0.96 LR	0.96 LR	0.96 LR	0.87 LR	0.88 LR	0.89 LR
OCT values	0.65 LSTM	0.83 RNN, LSTM	0.85 LSTM	0.56 LSTM	0.83 LSTM	0.80 RNN
OCT values + images	0.95 LSTM, GRU	0.95 RNN, LSTM, GRU	0.98 LSTM	0.81 GRU	0.87 LSTM	0.81 LSTM
OCT values + images + VA	0.98 RNN	0.98 LSTM	0.99 LSTM	0.90 RNN	0.94 LSTM	0.87 LSTM

## Data Availability

Please contact Simona Delia Nicoara for the OCT images dataset.
